# Cumulative fluid accumulation is associated with the development of acute kidney injury and non-recovery of renal function: a retrospective analysis

**DOI:** 10.1186/s13054-019-2673-5

**Published:** 2019-12-03

**Authors:** Jing Zhang, Siobhan Crichton, Alison Dixon, Nina Seylanova, Zhiyong Y. Peng, Marlies Ostermann

**Affiliations:** 1 Department of Critical Care, King’s College London, Guy’s and St Thomas’ NHS Foundation Trust, London, SE1 7EH UK; 2 Department of Critical Care Medicine, Zhongnan Hospital of Wuhan University, Wuhan, China; 3 Medical Research Council Clinical Trials Unit, University College London, London, UK; 4 Department of Critical Care, Guy’s and St Thomas’ NHS Foundation Trust, London, UK; 5 Sechenov Biomedical Science and Technology Park, Sechenov First Moscow State Medical University, Moscow, Russian Federation

**Keywords:** Acute kidney injury, Recovery, Cumulative fluid balance

## Abstract

**Background:**

Acute kidney injury (AKI) is common in patients in the intensive care unit (ICU) and may be present on admission or develop during ICU stay. Our objectives were (a) to identify factors independently associated with the development of new AKI during early stay in the ICU and (b) to determine the risk factors for non-recovery of AKI.

**Methods:**

We retrospectively analysed prospectively collected data of patients admitted to a multi-disciplinary ICU in a single tertiary care centre in the UK between January 2014 and December 2016. We identified all patients without AKI or end-stage renal failure on admission to the ICU and compared the outcome and characteristics of patients who developed AKI according to KDIGO criteria after 24 h in the ICU with those who did not develop AKI in the first 7 days in the ICU. Multivariable logistic regression was applied to identify factors associated with the development of new AKI during the 24–72-h period after admission. Among the patients with new AKI, we identified those with full, partial or no renal recovery and assessed factors associated with non-recovery.

**Results:**

Among 2525 patients without AKI on admission, the incidence of early ICU-acquired AKI was 33.2% (AKI I 41.2%, AKI II 35%, AKI III 23.4%). Body mass index, Sequential Organ Failure Assessment score on admission, chronic kidney disease (CKD) and cumulative fluid balance (FB) were independently associated with the new development of AKI. By day 7, 69% had fully recovered renal function, 8% had partial recovery and 23% had no renal recovery. Hospital mortality was significantly higher in those without renal recovery. Mechanical ventilation, diuretic use, AKI stage III, CKD, net FB on first day of AKI and cumulative FB 48 h later were independently associated with non-recovery with cumulative fluid balance having a U-shape association.

**Conclusions:**

Early development of AKI in the ICU is common and mortality is highest in patients who do not recover renal function. Extreme negative and positive FB were strong risk factors for AKI non-recovery.

## Background

Acute kidney injury (AKI) is common during critical illness, affecting > 50% of patients in the intensive care unit (ICU) [1]. It is a syndrome rather than a defined diagnosis, has many different aetiologies and can develop at different stages throughout critical illness [2]. There is increasing evidence that AKI is associated with short- and long-term complications and high healthcare costs [3]. Patients who leave hospital alive remain at risk of chronic health problems, including chronic kidney disease (CKD) and end-stage renal disease (ESRD) [4, 5]. The risk is particularly high in patients with more severe, recurrent or prolonged AKI and in those with reduced renal functional reserve.

Many patients already suffer from AKI when admitted to the ICU but a large proportion develop AKI later whilst receiving critical care [6]. Their risk of dying is significantly higher than that of patients with AKI on admission. There is a clear need for better strategies to prevent the development of AKI during critical illness and to reduce the risk of progression in those who develop AKI. Although some risk factors are not modifiable, for instance advanced age or pre-existing CKD, others are avoidable or potentially amenable to modification, for instance exposure to nephrotoxic drugs [7–9]. A better understanding of the risk of ICU-acquired AKI and the identification of potentially modifiable risk factors is essential to reduce the global burden of AKI.

The objectives of this study were (a) to identify factors associated with the development of new AKI during early stay in the ICU and (b) to ascertain risk factors for non-recovery of AKI.

## Methods

### Setting

Guy’s and St Thomas’ NHS Foundation Hospital is a tertiary care centre with a 54-bed consultant-led multi-disciplinary ICU. The ICU has a fully computerized electronic patient record system where all data are recorded at the time of generation.

### Patients and study design

We analysed the departmental database containing prospectively collected data of adult patients (≥ 18 years) admitted to the ICU between January 2014 and December 2016 and identified all patients with AKI as defined by the urine and creatinine criteria of the Kidney Disease Improving Global Outcome (KDIGO) classification [9]. In cases where baseline renal function was not available, we retrospectively imputed the serum creatinine using the Modification of Diet in Renal Disease (MDRD) equation, as suggested by the KDIGO expert group [9].

In our ICU, serum creatinine is measured on admission to the ICU, routinely every morning at around 6 a.m. and in-between as directed by the clinical team. Urine output is monitored and recorded hourly. To identify factors associated with the new development of AKI, we only analysed patients who did not have AKI in the first 24-h time period after ICU admission [10]. Therefore, we excluded patients with (i) AKI on admission or within the first 24 h of admission to the ICU, (ii) known ESRD, (iii) a renal transplant, (iv) renal replacement therapy (RRT) prior to ICU admission and (v) admission to the ICU after a nephrectomy. Other exclusion criteria were previous admission to the ICU during the same hospitalization, ICU stay < 48 h and pregnancy. We compared patients who developed AKI between the 24–72-h time period after admission to the ICU with patients who did not develop AKI within 7 days after admission to the ICU.

Among patients with AKI acquired in the ICU, we determined the degree of renal recovery at day 7 after ICU admission and distinguished between (a) full renal recovery (i.e. return of serum creatinine to baseline and urine output > 0.5 ml/kg), (b) partial renal recovery (i.e. improvement of AKI to lower stage but no return to baseline) and (c) no renal recovery. Patients who were receiving RRT on day 7 were classified as having “no renal recovery”; patients who had received RRT in the first 6 days in the ICU and were no longer on RRT on day 7 were classified as having “partial recovery” independent of serum creatinine concentration. We also collected serum creatinine results at hospital discharge and investigated a change from baseline.

### Data collection

We extracted the following data: demographics, chronic co-morbidities, Sequential Organ Failure Assessment (SOFA) score on admission to the ICU and on the first day of AKI, daily haemodynamic variables and serum creatinine for 7 days after admission to the ICU and at hospital discharge. Daily cumulative fluid balance was calculated automatically in our electronic medical records and defined as the difference between the total fluid intake from all sources (intravenous fluids, blood products, enteral and parenteral nutrition and medications) and total output (urine, effluent, output from drains and gastrointestinal losses). We recorded daily cumulative fluid balance in millilitres/24 h and percentage body weight (BW %). We extracted data on the type of organ support, use of diuretics and exposure to specific nephrotoxic medications (aminoglycosides, non-steroidal anti-inflammatory drugs, including paracetamol, chemotherapy, contrast, vancomycin and antiretroviral drugs).

In the cohort of patients who developed new AKI during the 24–72-h period in the ICU, we identified the median day of AKI development and used this day as the reference point for comparison with the non-AKI group. To identify risk factors for non-recovery of renal function, we distinguished between factors prior to the development of new AKI and factors post-onset of AKI. The main outcomes were the development of new AKI, renal function on day 7 or day of hospital discharge (whatever occurred first) and survival status at the ICU and hospital discharge.

### Statistical analysis

Continuous variables were summarized as mean and standard deviation (SD) or median and interquartile range (IQR) and compared between those who did and did not develop AKI in the first 24–72 h in the ICU, and those who did and did not recover from AKI, using the independent-samples *t* test or Mann-Whitney *U* test, as appropriate. Categorical variables were summarized as frequencies and percentages and compared using the chi-squared test. In the first multivariable model, the relationships between odds of developing AKI and demographic and clinical characteristics significant in univariable analyses were explored using logistic regression. Variables with small sample sizes (e.g. epinephrine use) and variables that were highly colinear with other variables (e.g. CKD and baseline serum creatinine) were not included in the multivariable analyses. Fractional polynomials were used to model the non-linear relationship between fluid balance and risk of AKI. In patients with AKI, multivariable logistic regression models were also used to explore the relationship between odds of non-recovery and (a) variables known on the first day of AKI only and (b) variables representing conditions after the development of AKI only. Fractional polynomials were again used to explore the effect of cumulative fluid balance in model (1) and net fluid balance on day of AKI (2). Survival analysis was used to describe cumulative hospital survival. *p* values < 0.05 were considered statistically significant. Statistical analyses were performed using IBM SPSS Statistics 20.0 and STATA 15/IC.

## Results

**Fig. 1:**
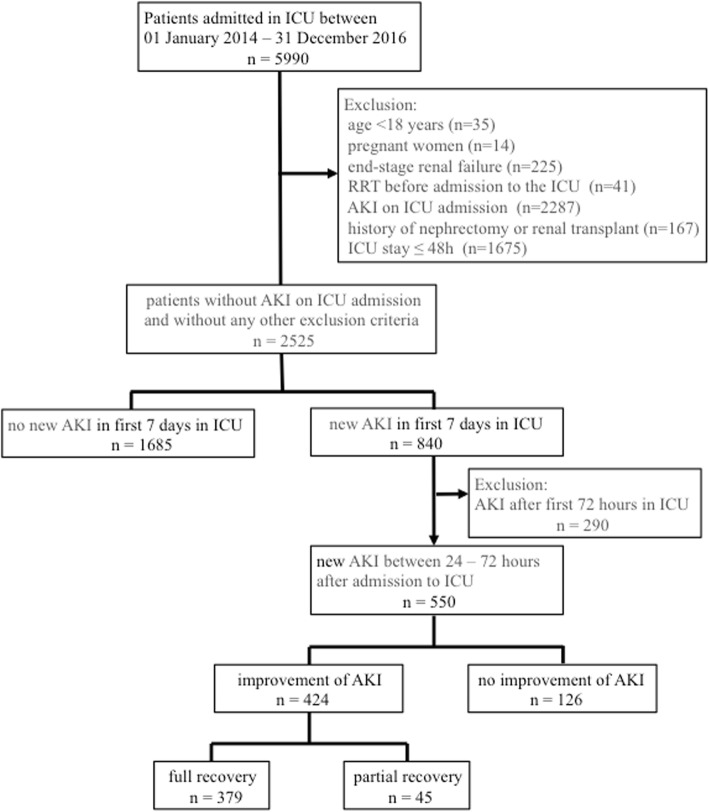
Patient flow. AKI acute kidney injury, ICU intensive care unit, RRT renal replacement therapy

Between January 2014 and December 2016, 5990 patients were admitted to the ICU; 2525 patients did not have AKI on admission and did not meet any exclusion criteria (Fig. 1). Among this cohort, 840 (33%) patients developed new AKI at median day 3 (IQR 2–4) compared to 1685 (67%) patients who did not develop AKI during the first 7 days in the ICU. The majority of patients with new AKI had AKI stage I (41%) followed by AKI stage II (35%) and AKI stage III (24%). Patients who developed new AKI were older and characterized by a significantly higher SOFA score and higher CVP on admission to the ICU, a higher prevalence of pre-existing CKD and cardiovascular disease, greater need for advanced organ support, higher cumulative fluid balance and longer periods of inotrope and/or vasopressor support compared to patients without new AKI. (Table 1).

**Table 1:** Demographic and baseline clinical parameters

Variables*	All patients without AKI on admission (*n* = 2525)	New AKI in the ICU (*n* = 840)	No new AKI in the ICU (*n* = 1685)	*p* value
Demographics				
Age	62 [48, 74]	64 [51, 74]	61 [43, 72]	0.02
Male sex	1498 (59.3)	519 (62.0)	979 (58.0)	0.06
BMI	24.2 [23, 28.1]	25.4 [23.5, 30.0]	24.2 [22.9, 29.0]	<0.001
Body weight [kg]	70 [63, 81]	73 [65, 89]	70 [60, 78]	< 0.001
Parameters on the day of ICU admission				
Medical admission	2012 (80)	677 (81)	1335 (79)	0.69
Elective surgical admission	367 (15)	113 (14)	254 (16)	
Emergency surgical admission	146 (5)	50 (6)	96 (6)	
Lowest MAP [mmHg]	59 [53, 64]	56 [51, 61]	59 [55, 62]	0.01
SOFA score	5 [3, 7]	5 [3, 8]	4 [3, 7]	<0.001
CVP [mmHg]	12 [8, 17]	13 [9, 18]	11 [7, 16]	<0.001
Comorbidities				
Baseline serum creatinine [μmol/L)	83 [63, 113]	100 [75, 140]	81 [60, 117]	<0.001
Chronic kidney disease	228 (9.0)	111 (13.3)	117 (6.9)	<0.001
Chronic lung disease	715 (28.3)	246 (29.4)	469 (27.8)	0.40
Chronic liver disease	1645 (65.1)	553 (66.1)	1092 (67.1)	0.51
Cardiovascular disease	545 (21.6)	222 (26.5)	343 (20.3)	<0.001
Congestive heart failure	200 (8.0)	80 (9.6)	120 (7.1)	0.04
Diabetes mellitus	517 (20.5)	197 (23.5)	320 (19.0)	0.01
Cerebrovascular disease	206 (8.2)	73 (8.7)	133 (7.9)	0.49
Cancer	730 (28.9)	218 (26.0)	512 (30.3)	0.03
Primary diagnostic code for ICU admission				
Respiratory	899 (35.6)	298 (35.6)	601 (35.6)	1.00
Neurologic	179 (7.1)	61 (7.3)	118 (7.0)	0.81
Post-surgery	429 (17.0)	132 (15.8)	297 (17.6)	0.26
Cardiovascular	391 (15.4)	145 (17.3)	246 (14.6)	0.08
Gastrointestinal	183 (7.2)	59 (7.0)	124 (7.3)	0.81
Urinary	57 (2.3)	14 (1.7)	43 (2.5)	0.20
Sepsis	210 (8.3)	84 (10.0)	126 (7.5)	0.03
Other	339 (13.4)	109 (13.0)	230 (13.6)	0.71
Organ support from ICU admission to the day of AKI or day 3				
Mechanical ventilation	1444 (57.2)	512 (61.2)	932 (55.2)	0.04
ECMO	130 (5.1)	61 (7.3)	69 (4.1)	0.01
IABP	62 (2.5)	31 (3.7)	31 (1.8)	0.01
Surgery	101 (4.0)	35 (4.2)	66 (3.9)	0.75
Epinephrine	27 (1.1)	13 (1.6)	14 (0.8)	0.10
Norepinephrine	1050 (41.6)	427 (50.8)	627 (37.1)	<0.001
Vasopressin	9 (0.4)	5 (0.6)	4 (0.2)	0.17
Time (in the first 7 days) spent on inotropic and vasoactive medications				
Number of days on at least 1 inotrope or vasopressor	0 [0, 0]	1 [0, 3]	0 [0, 2]	< 0.001
0 days	1405 (56)	382 (45)	1023 (61)	
1–3 days	725 (29)	252 (30)	473 (28)	
4 or more days	395 (16)	206 (25)	189 (11)	
Number of days on > 1 inotrope or vasopressor	0 [0, 0]	0 [0, 0]	0 [0, 0]	< 0.001
0 days	2324 (92)	737 (88)	1587 (94)	
1–3 days	178 (7)	87 (10)	91 (5)	
4 or more days	23 (1)	16 (2)	7 (0)	
Number of days on at least 1 vasopressor	0 [0, 0]	1 [0, 3]	0 [0, 2]	< 0.001
0 days	1428 (57)	386 (46)	1042 (62)	
1–3 days	718 (28)	251 (30)	467 (28)	
4 or more days	379 (15)	203 (24)	176 (11)	
Number of days on > 1 vasopressor	0 [0, 0]	0 [0, 0]	0 [0, 0]	0.001
0 days	2502 (99)	825 (98)	1677 (99)	
1–3 days	23 (1)	15 (2)	8 (1)	
4 or more days	0	0	0	
Potentially nephrotoxic exposures				
Vancomycin	240 (9.5)	82 (9.8)	158 (9.4)	0.72
Diuretic	1185 (46.9)	381 (45.5)	804 (47.6)	0.33
Aminoglycosides	899 (35.6)	291 (34.8)	608 (36.0)	0.57
ACE-I/ARB	180 (7.1)	67 (8.0)	113 (6.7)	0.25
Contrast	252 (10.0)	88 (10.5)	164 (9.7)	0.53
Chemotherapy	42 (1.7)	11 (1.3)	31 (1.8)	0.88
Antiretroviral drugs	40 (1.6)	11 (1.3)	30 (1.8)	0.41
NSAID	51 (2.0)	8 (1.0)	43 (2.5)	0.01
Fluid balance on the day of AKI/day 3				
Cumulative FB in ml	1389 (3455)	2148.4 (3667.5)	1002 (3276)	<0.001
% of fluid balance in % BW	1.66 [−0.7, 4.3]	3.98 [1.2, 6.9]	2.29 [−0.07, 5.14]	<0.001
Outcomes				
ICU stay (days)		13 [9, 21]	9 [5, 17]	< 0.01
Hospital stay (days)		31 [19, 56]	22 [12, 43]	0.94
ICU mortality (%)		175 (20.9)	94 (5.6)	< 0.01
Hospital mortality (%)		273 (35.4)	243 (15.4)	< 0.01

*ACE-I* angiotensin converting enzyme inhibitor, *ARB* angiotensin receptor blocker, *AKI* acute kidney injury, *BMI* body mass index, *BW* body weight, *CVP* central venous pressure, *ECMO* extracorporeal membrane oxygenation, *FB* fluid balance, *IABP* intra-aortic balloon pump, *ICU* intensive care unit, *MAP* mean arterial pressure, *NSAID* non-steroidal anti-inflammatory drug, *SD* standard deviation, *SOFA* Sequential Organ Failure Assessment

*Results displayed as *n* (%), mean (SD) or median [interquartile range]

Among all patients with new AKI, 550 patients developed AKI during the 24- to 72-h period after admission to the ICU. Their ICU mortality was 19.8% and hospital mortality was 32.2%. Among this cohort, 379 (69%) had full recovery of renal function during the 7-day period after admission to the ICU, 45 (8%) had partial renal recovery, and in 126 (23%) patients, renal function did not improve (Fig. 1 and Additional file 1). Patients without renal recovery were characterized by a higher SOFA score and higher CVP prior to the development of AKI and in the following 24–48 h (Additional file 1 and Additional file 2). In addition, a higher proportion of AKI patients with non-recovery were treated with extracorporeal membrane oxygenation (ECMO), mechanical ventilation and/or noradrenaline following the development of AKI suggesting that they had more associated organ failure. The risk of non-recovery was higher in patients with AKI stage III (Additional file 2). Finally, among 177 patients with new AKI who died in the hospital, 71 (40%) had no recovery of renal function compared to 56 of 373 patients (15%) with new AKI who were discharged from the hospital alive.

### Multivariable analyses

Multivariable logistic regression analysis showed that BMI, SOFA score on admission to the ICU, pre-existing CKD and cumulative FB on day of AKI or day 3 were independently associated with new AKI (Table 2). In a separate model including cumulative fluid balance in the first 24 h, fluid balance was not associated with risk of developing AKI (OR = 0.98, 95% CI, 0.96–1.01, *p* = 0.159).

**Table 2:** Multivariate analysis of risk factors for the development of new acute kidney injury

Variables	OR	95% CI for OR		*p* value
		Lower	Upper	
Age	1.00	1.00	1.01	0.24
BMI	1.06	1.04	1.07	<0.001
SOFA score on admission to the ICU	1.04	1.00	1.08	0.05
Lowest MAP	1.00	0.99	1.01	0.84
Sepsis	1.11	0.80	1.53	0.53
Chronic kidney disease	1.81	1.34	2.45	<0.001
Atherosclerotic cardiovascular disease	1.13	0.90	1.40	0.29
Congestive heart failure	1.12	0.81	1.56	0.50
Diabetes mellitus	1.06	0.85	1.33	0.60
Cancer	0.84	0.69	1.03	0.10
Mechanical ventilation	0.94	0.77	1.17	0.60
Norepinephrine use	1.20	0.94	1.54	0.15
NSAID use	0.48	0.22	1.05	0.07
Cumulative fluid balance on the day of AKI or day 3 in the ICU	1.11	1.08	1.14	<0.001

Model diagnostics:

There was no evidence of lack of fit (Hosmer Lemeshow *p* = 0.209) that the model was incorrectly specified (link test *p* = 0.300) or of multicollinearity among the included covariates (all variance inflation factors (VIF) < 1.5). There was no evidence of a non-linear relationship between cumulative fluid balance and AKI

*BMI* body mass index, *CI* confidence interval, *MAP* mean arterial pressure, *NSAID* non-steroidal anti-inflammatory drug, *SOFA* Sequential Organ Failure Assessment, *OR* odd ratio

**Fig. 2:**
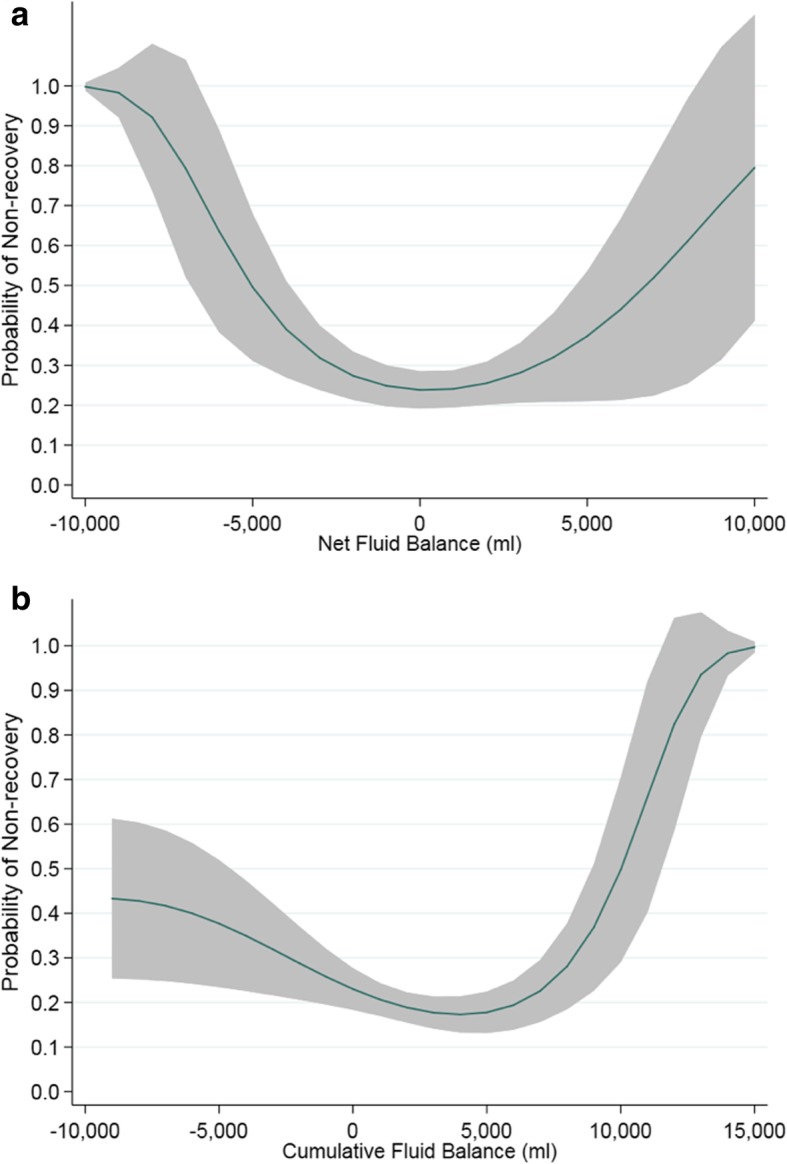
Association between fluid balance and non-recovery of renal function. **a** Association between net fluid balance on the first day of AKI and probability of renal non-recovery. **b** Association between cumulative fluid balance at 48 h after the onset of AKI and probability of renal non-recovery

In patients with new AKI, risk factors for non-recovery within the first 7 days included factors before and after the onset of new AKI. Independent pre-AKI risk factors for non-recovery were CKD, need for mechanical ventilation, use of diuretics and net FB on the first day of AKI (Table 3 and Fig. 2a). Severity of AKI, mechanical ventilation and cumulative FB in the 48-h period after the day of AKI were independent post-AKI risk factors for non-recovery of renal function (Table 3 and Fig. 2b).

**Table 3:** Multivariate analysis for risk of non-recovery after acute kidney injury

Variables	MVR (variables pre-AKI)^1^				MVR (variables post-onset of AKI)^2^			
	OR	95% CI for OR		*p* value	OR	95% CI for OR		*p* value
		Lower	Upper			Lower	Upper	
AKI stage					1			< *0.001*
AKI stage 2					0.63	0.31	1.28	0.21
AKI stage 3					2.24	1.16	4.31	*0.02*
SOFA score on admission to the ICU	1.05	0.96	1.15	0.26				
SOFA score on the day of AKI/day 3	–	–	–	–	1.10	0.99	1.22	0.09
Lowest MAP on the day of AKI	–	–	–	–	0.99	0.96	1.02	0.36
Chronic kidney disease	2.01	1.12	3.58	*0.02*	2.82	1.37	5.78	*0.01*
Reason for admission: respiratory	1.52	0.94	2.45	0.09				
Mechanical ventilation	2.29	1.24	4.26	*0.01*	4.34	2.05	9.15	< *0.001*
Norepinephrine use	1.21	0.64	2.30	0.56	0.56	0.25	1.26	0.16
Vancomycin use	1.10	0.48	2.48	0.83	1.40	0.65	3.01	0.39
Diuretic use	1.89	1.20	2.97	*0.01*				
Aminoglycoside use	1.13	0.69	1.83	0.63				

Model diagnostics:

There was no evidence of lack of fit (Hosmer Lemeshow *p* = 0.217 and 0.248) that the model was incorrectly specified (link test *p* = 0.082 and 0.345) or of multicollinearity among the included covariates (all variance inflation factors < 3) for model MVR 1 or MVR 2

*AKI* acute kidney injury, *CI* confidence interval, *MAP* mean arterial pressure, *ICU* intensive care unit, *MVR* multivariate analysis, *NSAID* non-steroidal anti-inflammatory drug, *SOFA* Sequential Organ Failure Assessment, *OR* odd ratio

^1^Also adjusted for the non-linear association between fluid balance and non-recovery (*p* < 0.001)

^2^Also adjusted for the non-linear association between net fluid balance and non-recovery (*p* = 0.016)

Both net FB and cumulative FB showed an independent U-shape association with renal recovery (Fig. 2a and b). In sensitivity analyses in which models were fitted separately to those who died in the hospital and those discharged alive, the U-shape relationship was present in both groups (Additional file 5: Figure S1 and Additional file 6: Figure S2). There were 37 patients with a cumulative FB of − 5 l or more on the day of AKI or day 3 in the ICU (in patients without AKI); they were less likely to be male, more likely to have congestive heart failure, chronic lung disease, a respiratory diagnosis, to be on ECMO and to have received a diuretic than those with cumulative FB less than − 5 l (Additional file 3). There were also 271 patients with a cumulative FB greater than + 5 l which was associated with lower MAP, higher baseline serum creatinine, known cardiovascular disease, gastrointestinal and sepsis diagnoses on admission and the use of norepinephrine.

### Outcomes

Hospital mortality was significantly higher in patients who developed AKI in the ICU compared to those without AKI (35.4% versus 15.4%, *p* = 0.01) (Table 1). Among AKI patients, hospital mortality was lowest in those who had full renal recovery in the first 7 days (22.8%) compared to patients with partial renal recovery (59.5%) or no recovery (64%) (Additional file 4 and Additional file 7: Figure S3). AKI patients also had a longer length of stay in the ICU, especially if renal function did not recover.

## Discussion

This retrospective analysis demonstrates that ICU-acquired AKI is common and associated with a high risk of death, especially if renal function does not recover quickly. More than 30% of critically ill patients who did not have AKI on admission to the ICU developed AKI during the first 24 to 72 h in the ICU. Pre-existing CKD, more severe AKI, need for mechanical ventilation and diuretic use were independently associated with a higher risk of non-recovery. Cumulative fluid balance was strongly associated with both the development of AKI and non-recovery. For non-recovery of renal function, the association was U-shaped; both extremely negative and extremely positive cumulative fluid balance were harmful to the kidney function. Finally, the chances of being discharged alive from the hospital were significantly better in patients who either did not develop AKI or recovered renal function within 1 week compared to those who still had AKI on day 7.

Although it is not always possible to modify risk factors for AKI, many episodes of AKI are considered to be preventable. It is therefore essential to identify those risk factors that can either be avoided or altered. In our analysis, cumulative fluid balance emerged as a strong risk factor with a U-shape association. Both inadequate fluid administration and fluid accumulation were independently associated with an increased risk of non-recovery. Cumulative fluid balance by the day of AKI (or day 3 in the ICU) was also strongly associated with the development of new AKI. Raimundo et al. previously showed that increased fluid administration in early AKI was an independent risk factor for progression to AKI stage III [11]. Data from the Sepsis Occurrence in Acutely Ill Patients demonstrated a 54% adjusted risk of death at 60 days in patients with AKI and a mean positive fluid balance [12]. Although the exact reasons for the deleterious impact of fluid overload on renal function are not known, obstruction of capillary blood flow and lymphatic drainage, renal congestion and impaired tissue oxygenation are likely to play an important role [13, 14]. These effects are particularly pronounced in encapsulated organs such as the liver and kidneys that lack the capacity to accommodate extra volume without an increase in the interstitial pressure. Importantly, there is no evidence that fluid accumulation is beneficial or necessary in AKI or during acute illness. Van Biesen et al. previously showed that additional fluid loading not only failed to improve renal function but was also associated with worsening respiratory function [15].

Fluid management is a key component of prevention and management of AKI [9, 16]. Although we showed that both negative and positive fluid balance were associated with worse outcomes, it is important to note that our study and others in the literature only demonstrate associations but do not prove a causal relationship [11, 12, 17]. It is certainly possible that there was more haemodynamic instability and hypotension among those who received larger volumes of fluid and accumulated more fluid and that haemodynamic instability per se may have contributed to the risk of AKI. The role of active fluid restriction in AKI with focus on the prevention of fluid overload has not been studied prospectively. However, data from the CLASSIC feasibility study showed that a protocol of restricting fluid administration after initial resuscitation in patients with septic shock was safe and associated with less worsening of AKI compared to standard care [18]. Until more data are available, our results, together with reports in the literature suggest that regular assessment of both daily fluid balance and cumulative fluid balance are crucial. Whilst fluid administration is an important component of the initial resuscitation phase and serves to prevent AKI in patients with intravascular hypovolaemia, there comes a point when fluids have a deleterious effect on renal function. Unfortunately, the exact timing and how best to identify the time point remain unclear.

Another interesting result of our analysis was the emergence of obesity as an independent risk factor for the development of AKI. Previous studies showed a higher risk of AKI in trauma and acute lung injury patients with higher BMI [19–21]. Others pointed out that the association between BMI and risk of AKI appeared to be a “U”-shaped curve with both under- and overweight patients being particularly at risk [22]. Billings et al. speculated that the association between obesity and AKI after cardiac surgery might be mediated by oxidative stress [23]. It is also possible that inappropriate dosing of nephrotoxic drugs in patients with a higher BMI plays a role. However, it is also important to acknowledge that the utilization of weight-based urine volume criteria may overestimate the risk of AKI in patients with a higher BMI resulting in an overestimation of AKI [24].

To the best of our knowledge, this study is the first to describe the non-linear relationship of cumulative FB and non-recovery of renal function. Both extremely negative and extremely positive FB were independently associated with non-recovery whilst increased FB was associated with the development of new AKI. These results should serve to underpin a future intervention trial exploring the role of fluid restriction in AKI.

Our analysis has some limitations which we would like to acknowledge. Due to the retrospective nature of the analysis, we were limited to the data that had been collected for clinical reasons. For instance, CVP measurements were not performed in all patients who developed AKI. As a result, we were not able to explore the relationship between CVP and AKI. Secondly, we defined ICU-acquired AKI as AKI that had developed at least 24 h after ICU admission. The decision was based on similar approaches in the literature [10]. However, we acknowledge that serum creatinine and urine output are relatively late indicators of AKI and that in a proportion of patients categorized with “AKI after ICU admission”, the onset of AKI may have been before ICU admission. In the future, the implementation of new AKI biomarkers may help to identify the onset of AKI more accurately and the AKI consensus definition and staging classification may be revised accordingly. Third, we excluded patients who developed AKI during the 72-h to 7-day period and acknowledge that they potentially had ICU-acquired AKI. The reason for excluding them was that we intended to analyse chances of renal recovery at day 7 and, therefore, we needed to allow patients with AKI a minimum time for kidney function to recover. Fourth, we evaluated renal recovery on day 7. The main reason was that we did not have complete data beyond this time period. We acknowledge that a proportion of patients may have either developed AKI later or recovered AKI after day 7. Fifth, in our models assessing factors associated with the development of new AKI, we included cumulative FB on the day of AKI (or day 3 in the ICU for those without AKI) and it is possible that the increased cumulative FB in those with AKI may be a consequence of AKI itself rather than a risk factor for AKI. Sixth, we used creatinine results without correcting for fluid balance. We acknowledge that AKI definition and classification could change with adjustment for fluid balance [25]. However, the current AKI consensus definition is based on creatinine values without correction. Finally, our data stem from a large multi-disciplinary ICU in a single centre. Therefore, our conclusions may not be generalizable to other patient populations.

## Conclusions

ICU-acquired AKI is associated with a high risk of mortality, especially if renal function does not recover by day 7. Cumulative fluid balance is a strong independent risk factor for the development and non-recovery of AKI with a U-shape relationship.

## Supplementary information

**Additional file 1:** Association between parameters before AKI and subsequent AKI recovery.

**Additional file 2:** Association between parameters during 24-48 hours period after AKI and AKI recovery.

**Additional file 3:** Comparison between patients with extremes of cumulative fluid balance on day of AKI or day 3 in ICU.

**Additional file 4: Table S1.** Outcomes in AKI with and without renal recovery

**Additional file 5: Figure S1.** Association between cumulative fluid balance on day of AKI and probability of non-recovery of renal function.

**Additional file 6: Figure S2.** Association between net fluid balance after 24 hours in ICU and probability of non-recovery of renal function.

**Additional file 7: Figure S3.** Survival curves of AKI and non-AKI patients.

## Data Availability

The datasets used and analysed during the current study are available from the corresponding author on reasonable request.

## Abbreviations

ACE-I: Angiotensin converting enzyme inhibitor
ARB: Angiotensin receptor blocker
AKI: Acute kidney injury
BMI: Body mass index
BW: Body weight
CI: Confidence interval
CKD: Chronic kidney disease
CRRT: Continuous renal replacement therapy
CVP: Central venous pressure
ECMO: Extracorporeal membrane oxygenation
ESRD: End-stage renal disease
FB: Fluid balance
IABP: Intra-aortic balloon pump
ICU: Intensive care unit
IQR: Interquartile ratio
KDIGO: Kidney Disease Improving Global Outcome
MAP: Mean arterial pressure
NSAID: Non-steroidal anti-inflammatory drug
REC: Research Ethics Committee
RRT: Renal replacement therapy
SD: Standard deviation
SOFA: Sequential Organ Failure Assessment
