# Supplementary material for: Cumulative fluid accumulation is associated with the development of acute kidney injury and non-recovery of renal function: a retrospective analysis
Source: Crit Care. 2019 Dec 3;23:392. doi: 10.1186/s13054-019-2673-5 (PMC6891953; doi:10.1186/s13054-019-2673-5)
Supplement: Supplementary file 1 — Additional file 1: Association between parameters before AKI and subsequent AKI recovery. [file 13054_2019_2673_MOESM1_ESM.docx]

**Additional File 1 Association between parameters before AKI and subsequent AKI recovery**

| **Variables *** |  | **AKI Non-recovery**  **(n = 126)** | **AKI recovery**  **(n = 424)** | |  |
| --- | --- | --- | --- | --- | --- |
|  |  |  | **partial recovery ( n = 45)** | **full recovery**  **(n =379)** | **p value** |
| **Demographics** | Age | 61 [49.5, 72.5] | 63.00 [54.5, 78.3] | 64 [51, 74] | 0.27 |
|  | Male sex | 85 (67.5) | 33 (73.3) | 236 (62.3) | 0.24 |
|  | BMI | 26.0 [24.0, 30.7] | 25.7 [23.4, 29.4] | 24.8 [23.0, 30.4] | 0.90 |
| **Parameters on ICU admission day** | Lowest MAP in mmHg | 60 [53.5, 63] | 58.5 [54, 67] | 60 [55, 65] | 0.06 |
|  | SOFA score | 8 [6, 10] | 7 [5.8,11.3] | 7 [5, 9] | ＜0.01 |
|  | CVP in mmHg | 15 [11, 21] | 14 [11,20] | 12 [8, 16] | ＜0.01 |
| **Comorbidities** | Baseline serum creatinine [μmol/L] | 110 [79, 171] | 96 [79, 135] | 90 [70, 126] | ＜0.01 |
|  | Chronic Kidney Disease | 27 (21.4) | 5 (11.1) | 49 (12.9) | 0.05 |
|  | Chronic Lung Disease | 31 (24.6) | 12 (26.7) | 108 (28.5) | 0.69 |
|  | Chronic Liver Disease | 88 (69.8) | 34 (75.6) | 237 (62.5) | 0.11 |
|  | Cardiovascular Disease | 37 (29.4) | 17 (37.8) | 98 (25.9) | 0.21 |
|  | Congestive Heart Failure | 13 (10.3) | 6 (13.3) | 37 (9.8) | 0.75 |
|  | Diabetes Mellitus | 32 (25.4) | 16 (35.6) | 91 (24.0) | 0.24 |
|  | Cerebrovascular Disease | 12 (9.5) | 7 (15.6) | 28 (7.4) | 0.16 |
|  | Cancer | 23 (18.3) | 9 (20.0) | 107 (28.2) | 0.06 |
| **Primary diagnostic code for ICU admission** | Respiratory | 48 (38.4) | 5 (11.1) | 114 (30.1) | ＜0.01 |
|  | Neurologic | 10 (7.9) | 5 (11.1) | 27 (7.1) | 0.63 |
|  | Post-surgery | 16 (12.7) | 9 (20.0) | 70 (18.5) | 0.29 |
|  | Cardiovascular | 27 (21.4) | 11 (24.4) | 69 (18.2) | 0.50 |
|  | Gastrointestinal | 11 (8.7) | 5 (11.1) | 25 (6.6) | 0.46 |
|  | Urinary | 1 (0.8) | 1 (2.2) | 11 (2.9) | 0.40 |
|  | Sepsis | 17 (13.5) | 6 (13.3) | 29 (7.7) | 0.10 |
|  | Other | 11 (8.7) | 6 (13.3) | 59 (15.6) | 0.16 |
| **Therapeutic intervention from ICU admission to AKI/ third day, n (%)** | Mechanical Ventilation | 100 (79.4) | 26 (57.8) | 210 (55.4) | ＜0.01 |
|  | ECMO | 28 (22.2) | 2 (4.4) | 10 (2.6) | ＜0.01 |
|  | IABP | 6 (4.8) | 3 (6.7) | 15 (4.0) | 0.68 |
|  | Surgery | 5 (4.0) | 1 (2.2) | 16 (4.2) | 0.81 |
|  | Epinephrine | 7 (5.6) | 2 (4.4) | 3 (0.8) | ＜0.01 |
|  | Norepinephrine | 86 (68.3) | 28 (62.2) | 169 (44.6) | ＜0.01 |
|  | Vasopressin | 4 (3.3) | 0 (0.0) | 1 (0.3) | 0.01 |
| **Potentially nephrotoxic exposures** | Vancomycin | 13 (10.3) | 9 (20.0) | 17 (4.5) | ＜0.01 |
|  | Diuretic | 60 (47.6) | 15 (33.3) | 115 (30.3) | ＜0.01 |
|  | Aminoglycosides | 49 (38.9) | 18 (40.0) | 104 (27.4) | 0.02 |
|  | ACE-I / ARB | 3 (2.4) | 2 (4.4) | 28 (7.4) | 0.11 |
|  | CT Scan or Angiography | 12 (9.5) | 2 (4.4) | 34 (9.0) | 0.56 |
|  | Chemotherapy | 0 (0.0) | 1 (2.2) | 6 (1.6) | 0.33 |
|  | Antiretroviral drugs | 1 (0.8) | 0 (0.0) | 6 (1.6) | 0.58 |
|  | Non-steroidal anti-inflammatory drugs | 1 (0.8) | 0 (0.0) | 4 (1.1) | 0.77 |
| **Fluid Management on the AKI/ third day** | Cumulative fluid balance in ml | 2515 [537, 4910] | 2390 [510, 4995] | 1870 [360, 3480] | 0.07 |
|  | Percentage of fluid balance (% of BW] | 4.07 [1.00, 8.66] | 4.88 [1.71, 7.05] | 3.79 [1.55, 6.32] | 0.08 |
| **Mortality** | ICU mortality | 48 (38.1%) | 20 (44.4%) | 41 (10.8%) | <0.01 |
|  | Hospital mortality | 72 (57.1%) | 26 (57.8%) | 81 (21.4%) | <0.01 |

* results displayed as n (%) or median [interquartile range]

Abbreviations: ACE-I = angiotensin converting enzyme inhibitor; ARB = angiotensin receptor blocker; AKI = acute kidney injury; BMI = body mass index; BW = body weight; CVP = central venous pressure; CT = computer tomography; ECMO = extracorporeal membrane oxygenation; FB = fluid balance; IABP = intra-aortic balloon pump; ICU = intensive care unit; MAP = mean arterial pressure; NSAID = non-steroidal anti-inflammatory drug; SD = standard deviation; SOFA = sequential organ failure assessment
