# Supplementary material for: Cumulative fluid accumulation is associated with the development of acute kidney injury and non-recovery of renal function: a retrospective analysis
Source: Crit Care. 2019 Dec 3;23:392. doi: 10.1186/s13054-019-2673-5 (PMC6891953; doi:10.1186/s13054-019-2673-5)
Supplement: Supplementary file 2 — Additional file 2: Association between parameters during 24-48 hours period after AKI and AKI recovery. [file 13054_2019_2673_MOESM2_ESM.docx]

**Additional File 2 Association between parameters during 24-48 hours period after AKI and AKI recovery**

| **Variables *** | | **AKI non-recovery**  **( n = 126)** | **AKI recovery**  **(n = 424 )** | |  |
| --- | --- | --- | --- | --- | --- |
|  |  |  | **partial recovery ( n = 45)** | **full recovery (n =379 )** | **p value** |
| **General condition after AKI 24 hours** | Lowest MAP in mmHg | 61 [56, 66] | 61 [57.3, 68.3] | 64 [58, 71] | 0.01 |
|  | CVP in mmHg | 15[11, 21] | 14.0 [10.8,19.5] | 12 [8,16] | ＜0.01 |
|  | SOFA score | 9 [5, 12] | 7.5 [6, 12] | 5 [3, 8] | ＜0.01 |
| **Comorbid disease** | Chronic Kidney Disease | 27 (21.4) | 5 (11.1) | 49 (12.9) | 0.05 |
|  | Chronic Lung Disease | 31 (24.6) | 12 (26.7) | 108 (28.5) | 0.69 |
|  | Chronic Liver Disease | 88 (69.8) | 34 (75.6) | 237 (62.5) | 0.11 |
|  | Cardiovascular Disease | 37 (29.4) | 17 (37.8) | 98 (25.9) | 0.21 |
|  | Congestive Heart Failure | 13 (10.3) | 6 (13.3) | 37 (9.8) | 0.75 |
|  | Diabetes Mellitus | 32 (25.4) | 16 (35.6) | 91 (24.0) | 0.24 |
|  | Cerebrovascular Disease | 12 (9.5) | 7 (15.6) | 28 (7.4) | 0.16 |
|  | Cancer | 23 (18.3) | 9 (20.0) | 107 (28.2) | 0.06 |
| **Therapeutic intervention during 24 hours period after AKI** | Mechanical Ventilation | 96 (80.0) | 22 (50.0) | 131 (40.8) | ＜0.01 |
|  | ECMO | 29 (24.4) | 2 (4.7) | 10 (3.4) | ＜0.01 |
|  | IABP | 4 (3.4) | 3 (7.0) | 9 (3.0) | 0.42 |
|  | Surgery | 1 (0.8) | 1 (2.3) | 4 (1.2) | 0.76 |
|  | Epinephrine | 2 (1.6) | 2 (4.4) | 2 (0.50 | 0.05 |
|  | Norepinephrine | 78 (61.9) | 24 (53.3) | 111 (29.3) | ＜0.01 |
|  | Vasopressin | 2 (1.7) | 0 | 0 | 0.05 |
| **Potentially nephrotoxic exposures** | Vancomycin | 18 (15.0) | 9 (20.5) | 21 (6.5) | ＜0.01 |
|  | Diuretic | 45(37.5) | 18 (40.9) | 118 (36.8) | 0.87 |
|  | Aminoglycosides | 33(27.5) | 13 (29.5) | 63 (19.6) | 0.11 |
|  | ACE-I / ARB | 3(2.5) | - | 15 (4.7) | 0.22 |
|  | Contrast | 6(5.0) | 2 (4.5) | 10 (3.1) | 0.62 |
|  | Chemotherapy | - | 1 (2.3) | 3 (0.9) | 0.34 |
|  | Antiretroviral drugs | 1 (0.8) | 1 (2.3) | 6 (1.9) | 0.71 |
|  | NSAID | - | 1 (2.3) | 3 (0.9) | 0.34 |
| **Fluid Management after AKI day 48 hours** | Cumulative fluid balance in ml | 44 {2201} | 760 {1259} | 130 {1100} | 0.01 |
|  | Percentage of fluid balance (% of BW] | 0.08 {2.36} | 1.00 {1.55} | 0.22 {1.54} | 0.01 |
| **Worst AKI stage** | I | 37 (29.4) | - | 177 (46.7) | ＜0.01 |
|  | II | 25 (19.8) | 24 (53.3) | 136 (35.9) |  |
|  | III | 64 (50.8) | 21 (46.7) | 66 (17.4) |  |

* results displayed as n (%) or median [interquartile range]

Abbreviations: ACE-I = angiotensin converting enzyme inhibitor; ARB = angiotensin receptor blocker; AKI = acute kidney injury; BMI = body mass index; BW = body weight; CVP = central venous pressure; ECMO = extracorporeal membrane oxygenation; FB = fluid balance; IABP = intra-aortic balloon pump; ICU = intensive care unit; MAP = mean arterial pressure; NSAID = non-steroidal anti-inflammatory drug; SD = standard deviation; SOFA = sequential organ failure assessment
