# Supplementary material for: Cumulative fluid accumulation is associated with the development of acute kidney injury and non-recovery of renal function: a retrospective analysis
Source: Crit Care. 2019 Dec 3;23:392. doi: 10.1186/s13054-019-2673-5 (PMC6891953; doi:10.1186/s13054-019-2673-5)
Supplement: Supplementary file 3 — Additional file 3: Comparison between patients with extremes of cumulative fluid balance on day of AKI or day 3 in ICU. [file 13054_2019_2673_MOESM3_ESM.docx]

**Additional File 3 Comparison between patients with extremes of cumulative fluid balance on day of AKI or day 3 in ICU**

| **Variables** | | **Cumulative FB < -5L**  **(n=37)** | **Cumulative FB -5 to +5l**  **(n=2161)** | **Cumulative FB>5l**  **(n=271)** | **p value** |
| --- | --- | --- | --- | --- | --- |
| **Demographics** | Age | 59 [44, 73] | 62 [48, 74] | 64 [51, 75] | 0.12 |
|  | Male sex | 15 (40.5) | 1282 (59.3) | 172 (63.5) | 0.03 |
|  | BMI | 25 [24, 32] | 24 [23, 28] | 24 [22, 28] | 0.09 |
|  | Weight | 70 [64, 81] | 70 [60, 83] | 70 [60, 77] | 0.83 |
| **Parameters on day of ICU admission** | lowest MAP [mmHg] | 58 [54, 64] | 60 [54, 64] | 56 [50, 60] | <0.001 |
|  | SOFA score | 3 [2, 7] | 5 [3, 7] | 4 [2, 7] | 0.27 |
|  | CVP [mmHg] | 11 [4, 14] | 12 [8, 17] | 14 [10, 19] | 0.003 |
| **Comorbidities** | Baseline serum creatinine [μmol/L) | 80 [51, 115] | 82 [52, 111] | 94 [70, 131] | <0.001 |
|  | Chronic kidney disease | 4 (11) | 194 (9) | 25 (9) | 0.91 |
|  | Chronic lung disease | 16 (43) | 628 (29) | 60 (22) | 0.008 |
|  | Chronic liver disease | 28 (76) | 1415 (65) | 170 (63) | 0.28 |
|  | Cardiovascular disease | 8 (22) | 461 (21) | 87 (32) | <0.001 |
|  | Congestive heart failure | 9 (24) | 170 (8) | 20 (7) | 0.001 |
|  | Diabetes mellitus | 10 (27) | 437 (20) | 61 (23) | 0.42 |
|  | Cerebrovascular disease | 2 (5) | 174 (8) | 25 (9) | 0.66 |
|  | Cancer | 8 (22) | 633 (29) | 68 (25) | 0.22 |
| **Primary diagnostic code for ICU admission** | Respiratory | 22 (59) | 804 (37) | 56 (21) | <0.001 |
|  | Neurologic | 1 (3) | 158 (7) | 15 (6) | 0.33 |
|  | Post-surgery | 1 (3) | 371 (17) | 49 (18) | 0.06 |
|  | Cardiovascular | 8 (22) | 325 (15) | 48 (18 | 0.30 |
|  | Gastrointestinal | 3 (8) | 139 (6) | 38 (14) | <0.001 |
|  | Urinary | 1 (3) | 50 (2) | 6 (2) | 0.98 |
|  | Sepsis | 1 (3) | 159 (7) | 43 (16) | <0.001 |
|  | Other | 3 (8) | 290 (13) | 39 (14) | 0.57 |
| **Organ support during period from ICU admission to day of AKI or day 3** | Mechanical ventilation | 16 (43) | 1210 (56) | 196 (72) | <0.001 |
|  | ECMO | 6 (16) | 111 (5) | 12 (4) | 0.009 |
|  | IABP | 0 | 47(2) | 14 (5) | 0.02 |
|  | Surgery | 1(3) | 63 (3) | 15 (6) | 0.07 |
|  | Epinephrine | 0 | 19 (1) | 6 (2) | 0.14 |
|  | Norepinephrine | 14 (38) | 835 (39) | 189 (70) | <0.001 |
|  | Vasopressin | 0 | 7 (0) | 2 (1) | 0.36 |
| **Potentially nephrotoxic exposures** | Vancomycin | 4 (11) | 176 (8) | 39 (14) | 0.003 |
|  | Diuretic | 27 (73) | 1020 (47) | 124 (46) | 0.007 |
|  | Aminoglycosides | 12 (32) | 647 (30) | 141 (52) | <0.001 |
|  | ACE-I / ARB | 7 (19) | 156 (7) | 15 (6) | 0.01 |
|  | Chemotherapy | 0 | 39 (2) | 2 (1) | 0.44 |
|  | Antiretroviral drugs | 0 | 32 (1) | 4 (2) | 0.10 |
|  | NSAID | 0 | 50 (2) | 1 (1) | 0.09 |

* results displayed as n (%) or median [interquartile range]

Abbreviations: ACE-I = angiotensin converting enzyme inhibitor; ARB = angiotensin receptor blocker; AKI = acute kidney injury; BMI = body mass index; BW = body weight; CVP = central venous pressure; ECMO = extracorporeal membrane oxygenation; FB = fluid balance; IABP = intra-aortic balloon pump; ICU = intensive care unit; MAP = mean arterial pressure; NSAID = non-steroidal anti-inflammatory drug; SD = standard deviation; SOFA = sequential organ failure assessment
