# Supplementary material for: Cumulative fluid accumulation is associated with the development of acute kidney injury and non-recovery of renal function: a retrospective analysis
Source: Crit Care. 2019 Dec 3;23:392. doi: 10.1186/s13054-019-2673-5 (PMC6891953; doi:10.1186/s13054-019-2673-5)
Supplement: Supplementary file 4 — Additional file 4: Table S1. Outcomes in AKI with and without renal recovery [file 13054_2019_2673_MOESM4_ESM.docx]

**Supplementary Table 4 Outcomes in AKI with and without renal recovery**

| **Variables *** | **no AKI (n=1685)** | **AKI (n = 840)** | **p value** | **AKI non recovery (n = 126)** | **AKI recovery (n = 424 )** | | **p value** |
| --- | --- | --- | --- | --- | --- | --- | --- |
|  |  |  |  |  | **partial recovery**  **(n = 45)** | **full recovery**  **(n = 379)** |  |
| **ICU stay (days)** | 13 [9, 21] | 9 [5, 17] | ＜0.01 | 15 [7, 21.5] | 7 [4, 14] | 6 [4, 12] | ＜0.01 |
| **Hospital stay (days)** | 31[19, 56] | 22 [12, 43] | 0.94 | 25 [15, 48] | 22 [9, 56] | 20[12, 35] | 0.11 |
| **ICU mortality** | 94 (5.6) | 175 (20.9) | ＜0.01 | 48 (38.1) | 20 (44.4) | 41 (10.8) | ＜0.01 |
| **Hospital mortality** | 243 (15.4) | 273 (35.4) | ＜0.01 | 71 (64.0) | 25 (59.5） | 81 (22.8) | ＜0.01 |

* results displayed as n (%) or median [interquartile range]

Abbreviations: AKI = acute kidney injury; ICU = intensive care unit
