# Supplementary figures and images for: Cumulative fluid accumulation is associated with the development of acute kidney injury and non-recovery of renal function: a retrospective analysis
Source: Crit Care. 2019 Dec 3;23:392. doi: 10.1186/s13054-019-2673-5 (PMC6891953; doi:10.1186/s13054-019-2673-5)

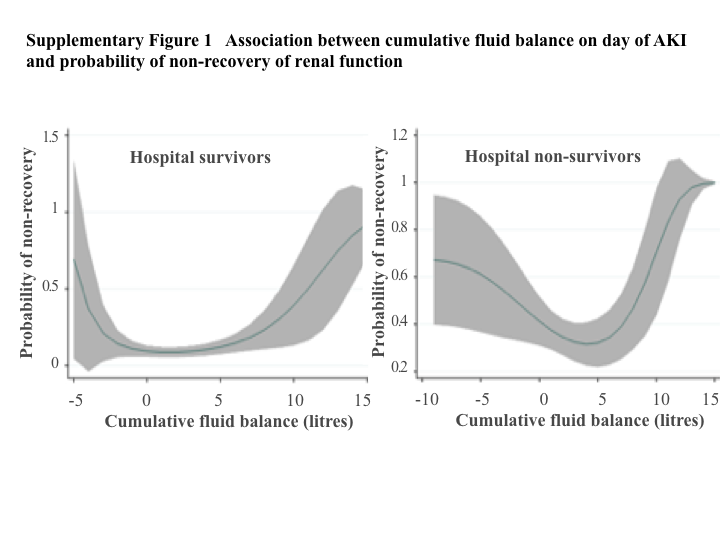

Supplement: Supplementary file 5 — Additional file 5: Figure S1. Association between cumulative fluid balance on day of AKI and probability of non-recovery of renal function. [file 13054_2019_2673_MOESM5_ESM.tiff]

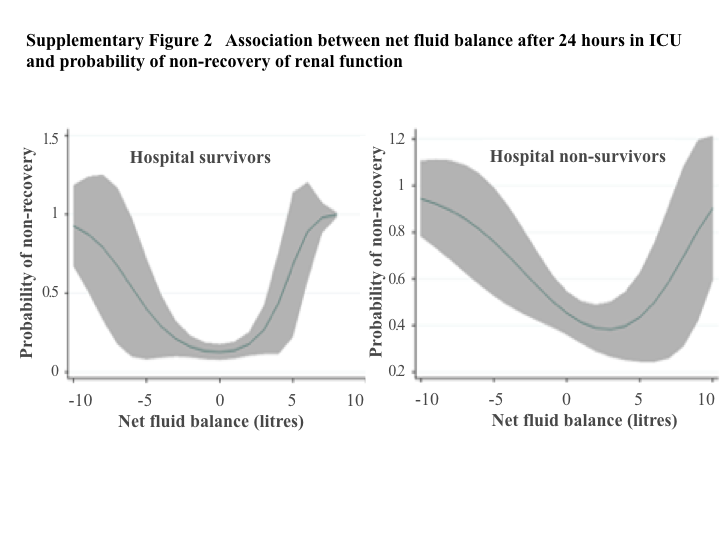

Supplement: Supplementary file 6 — Additional file 6: Figure S2. Association between net fluid balance after 24 hours in ICU and probability of non-recovery of renal function. [file 13054_2019_2673_MOESM6_ESM.tiff]

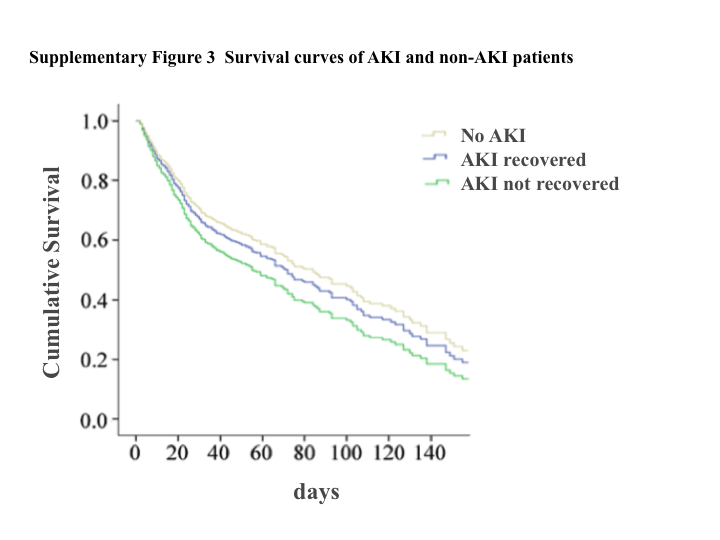

Supplement: Supplementary file 7 — Additional file 7: Figure S3. Survival curves of AKI and non-AKI patients. [file 13054_2019_2673_MOESM7_ESM.tiff]
